# The Road to a Cure: Emerging Treatments for Multiple Myeloma

**DOI:** 10.3390/cancers12123593

**Published:** 2020-12-01

**Authors:** Jo Caers

**Affiliations:** 1 Department of Clinical Hematology CHU De Liège, B-4000 Liège, Belgium; jo.caers@chu.ulg.ac.be; Tel.: +32-4-3667201; 2 Laboratory of Hematology, GIGA-I3, University of Liège, B-4000 Liège, Belgium

During 50 years of intensive research, we have learnt about the pathophysiology of multiple myeloma (MM) and improved the management of this disease. We understood that there are primary onco-genetic events that drive initiation and progression of a monoclonal plasma disorder that further interacts with the surrounding bone marrow micro-environment to become a malignant disorder [1]. Rather than following a linear process, this progression occurs through a branching pattern of clonal evolution [2]. From a clinical point of view, this multiclonal origin results in a heterogeneous disease that requires combination therapies to target different clones, each with a different sensitivity to the proposed molecules. First-line treatments actually contain triplet or even quadruplet schedules and obtain overall responses with high response rates of up to 100% after prolonged treatment, including high rates of minimal residual disease negativity [3]. 

The past decade saw an approval of next-generation molecules in different classes of anti-myeloma agents: proteasome inhibitors (ixazomib and carfilzomib), immuno-modulatory agents (pomalidomide), and monoclonal antibodies (elotuzumab and daratumumab) [4]. Other immunotherapeutic approaches are being developed and include bispecific antibodies and chimeric-antigen receptor (CAR) T cells [5]. Combining these agents further improved the second and third treatment lines. Unfortunately, MM becomes more and more resistant to the started treatments and patients often die because of a refractory disease. 

How can we tackle this disease and which are the current domains of MM research? Figure 1 illustrates the evolution of an MM patient, initially followed for a monoclonal gammopathy of undetermined significance (MGUS). He subsequently received different lines of treatment. After his first lines of treatment, an excellent response is seen, but as his disease progresses, the responses are less deep and not lasting. Current myeloma research tries to determine the genomic drivers underlying the initiation and progression of monoclonal gammopathies and to identify new strategies for early detection, prevention, and treatment of these diseases (Figure 1A). If we are able to offer a potent treatment that can cure these precursor diseases, the progression to overt myeloma can be prevented [6]. At diagnosis, we need accurate tools to identify high-risk disease and give detailed information on the extension of the disease [7]. A small portion of patients (10–15%) will progress early and such a resistance is associated with a very poor outcome (Figure 1B). More potent treatment combinations are needed for these patients and will probably combine next-generation immunotherapy and prolonged drug exposure. 

As mentioned in the previous paragraphs, the current schedules induce a clinical response in almost all patients and a complete response in 20% to 50% of patients [8,9]. In some of these patients, laboratory or imaging techniques can identify some (minimal) residual disease (Figure 1C). Understanding of the molecular features of this minimal residual disease (MRD) and identification of new targets expressed by these MRD cells would allow us to eradicate these residual cells and offer a another option for curing this malignancy [10]. Finally, new treatment strategies are required for patients presenting a refractory disease (Figure 1D). This can be achieved by selectively targeting proteins or signaling pathways relevant for the pathogenesis or drug resistance in individual patients. On the other hand, we need to continuously look for new treatment strategies that could offer prolonged disease control. 

This thematic issue “Emerging Treatment Strategies for Multiple Myeloma” offers a forum to present and publish original papers and review articles on current and future treatment strategies for MM. The hard work of thousands of researchers, each of them trying to solve a part of the puzzle, contributed to the observed improvement in the outcome of MM patients. Every finding brings us a bit closer to a cure for this disease. For this thematic issue, we are inviting you to submit papers on new immunotherapeutic strategies, signaling pathways, mechanisms of resistance, or new inhibitors that are in preclinical or clinical evaluation.

## Figures and Tables

**Figure 1:**
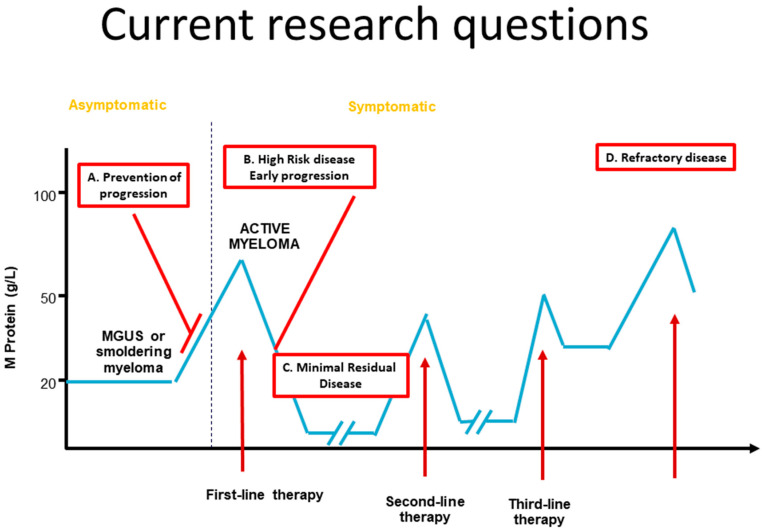
Current points of focus in the research field of multiple myeloma. The evolution of multiple myeloma can be tackled at different moments: (**A**) we can try to prevent the progression from a precursor disease to symptomatic myeloma, (**B**) we can better detect and treat high-risk patients, (**C**) we can try to eliminate the residual myeloma cells that persist after the initial treatment and (**D**) we should identify new potent treatment options for refractory patients.
